# Nursing diagnosis proposal “Impaired Peripheral Venous Return”: concept formation

**DOI:** 10.1590/0034-7167-2022-0426

**Published:** 2023-11-27

**Authors:** Hanna Priscilla da Silva Medeiros, Jéssica Naiara de Medeiros Araújo, Amanda Barbosa da Silva, Rodrigo Assis Neves Dantas, Camila Takáo Lopes, Allyne Fortes Vitor

**Affiliations:** I Universidade Federal do Rio Grande do Norte. Natal, Rio Grande do Norte, Brazil; II Universidade do Estado do Rio Grande do Norte. Caicó, Rio Grande do Norte, Brazil; III Universidade Federal de São Paulo. São Paulo, São Paulo, Brazil

**Keywords:** Venous Insufficiency, Lower Extremity, Nursing Diagnosis, Nursing Process, Concept Formation, Insuficiência Venosa, Extremidade Inferior, Diagnóstico de Enfermagem, Processo de Enfermagem, Formação de Conceito, Insuficiencia Venosa, Extremidad Inferior, Diagnóstico de Enfermería, Proceso de Enfermería, Formación de Concepto

## Abstract

**Objectives:**

to develop a nursing diagnosis proposal focused on venous return.

**Methods:**

this is a concept analysis according to the model proposed by Walker and Avant, which is operationalized through an integrative review. The study was carried out according to the Preferred Reporting Items for Systematic Reviews and Meta-Analyses protocol recommendations.

**Results:**

the analysis of the 131 studies allowed identifying attributes, antecedents and consequences. The most common attribute was decreased venous flow. The antecedents most frequently found were structural and/or functional valve deficiency, advanced age and peripheral venous thrombosis. The most common consequences were peripheral edema, venous ulcer and pain in the extremity.

**Conclusions:**

the formulated nursing diagnosis was proposed as part of Domain 4, Activity/rest, in Class 4, Cardiovascular/pulmonary responses, with eight defining characteristics, five related factors, six at-risk populations and four associated conditions.

## RESUMO

**Objetivos:**

desenvolver uma proposta de diagnóstico de enfermagem com foco no retorno venoso.

**Métodos:**

trata-se de uma análise de conceito segundo o modelo proposto por Walker e Avant, que é operacionalizado por meio de uma revisão integrativa. O estudo foi realizado de acordo com as recomendações do protocolo *Preferred Reporting Items for Systematic Reviews and Meta-Analyses*.

**Resultados:**

a análise dos 131 estudos permitiu a identificação de atributos, antecedentes e consequências. O atributo mais comum foi diminuição do fluxo venoso. Os antecedentes mais encontrados foram deficiência valvular estrutural e/ou funcional, idade avançada e trombose venosa periférica. As consequências mais comuns foram edema periférico, úlcera venosa e dor na extremidade.

**Conclusões:**

o diagnóstico de enfermagem formulado foi proposto como parte do Domínio 4, Atividade/repouso, na Classe 4, Respostas cardiovasculares/pulmonares, com oito características definidoras, cinco fatores relacionados, seis populações de risco e quatro condições associadas.

## RESUMEN

**Objetivos:**

desarrollar una propuesta de diagnóstico de enfermería centrada en el retorno venoso.

**Métodos:**

se trata de un análisis de concepto según el modelo propuesto por Walker y Avant, que se operacionaliza a través de una revisión integradora. El estudio se llevó a cabo de acuerdo con las recomendaciones del protocolo *Preferred Reporting Items for Systematic Reviews and Meta-Analyses*.

**Resultados:**

el análisis de los 131 estudios permitió identificar atributos, antecedentes y consecuencias. El atributo más común fue la disminución del flujo venoso. Los antecedentes encontrados con mayor frecuencia fueron deficiencia valvular estructural y/o funcional, edad avanzada y trombosis venosa periférica. Las consecuencias más comunes fueron edema periférico, úlcera venosa y dolor en la extremidad.

**Conclusiones:**

el diagnóstico de enfermería formulado fue propuesto como parte del Dominio 4, Actividad/reposo, en la Clase 4, Respuestas cardiovasculares/pulmonares, con ocho características definidoras, cinco factores relacionados, seis poblaciones de riesgo y cuatro condiciones asociadas.

## INTRODUCTION

Cardiovascular diseases (CVD) are the main cause of death worldwide. In 2016, 17.9 million people died from this type of disease, accounting for 31% of deaths globally^(1)^. Therefore, a 25% relative reduction in the risk of premature mortality from CVD is one of the global goals promoted by the World Health Organization (WHO)^(2)^.

Nevertheless, prevention and treatment of conditions related to impaired venous return/venous stasis (e.g., varicose veins, chronic venous insufficiency and venous thromboembolism) remain challenging factors. In the US, there were over 1.2 million cases of venous thromboembolism in 2016. In those with deep vein thrombosis (DVT), 4% have venous stasis ulcers. Varicose veins, a common manifestation of chronic venous insufficiency, were present in up to 23.3% in the San Diego population study. These conditions impair patients’ quality of life^(3-5)^.

Nurses have been engaged in the promotion of venous return from the lower limbs, in order to prevent undesirable outcomes, such as DVT^(6-8)^, stasis edema^(9)^ and venous ulcer recurrence^(10-11)^. However, the unique body of nursing knowledge contributing to the interdisciplinary approach in this clinical context might remain invisible, unless it is clearly articulated by nurses. The documentation of nursing diagnoses with a standardized terminology is a way to articulate nurses’ disciplinary knowledge^(12)^.

NANDA International (NANDA-I) in its 2018-2020 edition does not have specific nursing diagnoses focused on venous return. There are the two nursing diagnoses as follows: “Ineffective Tissue Perfusion” and “Excessive Fluid Volume”. However, it is noted that the diagnosis “Ineffective Tissue Perfusion” is sometimes inadvertently inferred to refer to all human circulatory responses. It is also noticed the use of “Excessive Fluid Volume” to all excessive entrance and/or retention of liquids in the organism. Thus, the inference of these diagnoses is directed to exclusively arterial characteristics, not contemplating individuals’ venous needs and fluid accumulation of all kinds, not specifying the peripheral fluid accumulation in question, respectively^(13)^.

In this sense, peripheral vascular disorders can lead to local and systemic complications. The availability of a nursing diagnosis related to these disorders within a widely known classification will support individual nursing assessment for clinical clues and possible predisposing, disabling, precipitating, or reinforcing factors that must be addressed to promote the best possible outcomes^(14-16)^.

The human responses represented by nursing diagnoses reflect the scientific development of the discipline. As nursing concepts and theoretical frameworks evolve, the hierarchical organization of concepts that are valid representations of disciplinary knowledge requires constant revisiting and refinement. NANDA-I encourages submissions of new evidence-based diagnostic proposals and reviews^(17)^. Therefore, having a proposal for a nursing diagnosis focused on venous return provides subsidies for developing independent nursing interventions and ensures the advancement of scientific nursing knowledge.

## OBJECTIVES

To develop a nursing diagnosis proposal focused on venous return.

## METHODS

### Ethical aspects

For this study, only the pertinent literature was used as a data source for the survey of attributes, antecedents and consequences of the concept. From this perspective, it was not submitted to a Research Ethics Committee because it is not a study involving human beings.

### Study design

This is a methodological study of concept analysis and diagnostic proposal based on the model proposed by Walker & Avant (2019)^(18)^, which is operationalized through an integrative literature review^(18)^. Concept analysis is equivalent to the first step to be performed in the process of validating nursing diagnoses. Identifying attributes, antecedents and consequences of a nursing diagnosis is the purpose of concept analysis for this study, which are used to identify the definition of diagnoses (attributes), related factors (antecedents) and defining characteristics (consequences). Furthermore, at this stage, at-risk populations and associated conditions that permeated the diagnosis are also identified^(15)^.

Walker and Avant’s concept analysis model includes the execution of eight steps, namely: 1) concept selection; 2) definition of the purpose of analysis; 3) identification of possible uses of the concept; 4) determination of critical or essential attributes; 5) construction of a model case; 6) construction of borderline, related, contrary and illegitimate cases; 7) identification of antecedents and consequences; and 8) definition of empirical referents. Steps 5 and 6 are optional to elucidate the concept attributes^(18)^. In the current study, steps 5 and 6 were not adopted, since the concept attributes were deemed clear enough.

The conceptual nucleus selected in this study was “Impaired Peripheral Venous Return”, with the aim of analyzing it as an undesirable human response in the context of nursing. The objectives of the analysis were to define the concept and identify the attributes, their antecedents and consequences.

In order to identify the possible uses of the concept and determine its critical, antecedent and consequential attributes, an integrative literature review was carried out, using the criteria established in the Preferred Reporting Items for Systematic Review and Meta-Analyses (PRISMA)^(19)^. This review was constructed according to the model described by Whittemore and Knafl (2005), structured in five methodological steps: identification of research questions; bibliographic search; data collection; critical analysis of data; and presentation and synthesis of results^(20)^.

### Study protocol/inclusion and exclusion criteria

The review was organized according to a previously elaborated research protocol for obtaining the sample, analysis and presentation of results. In order to prepare the guiding research question, the PCC (Population, Concept and Context) mnemonic strategy was applied^(21)^. In this study, it was adopted: P - adult patients; C - impaired peripheral venous return; C - cardiovascular clinical conditions/cardiovascular unit. Thus, the guiding research question based on the mnemonic was: what is the concept of impaired peripheral venous return in adult patients?

In order to contemplate the proposed objectives, subsequent research questions were elaborated, namely: what are the attributes that define peripheral venous return impairment? What are the antecedents and consequences of impaired peripheral venous return? What are the empirical references of clinical indicators of impaired peripheral venous return?

Data collection occurred by searching the following databases: Scopus (Elsevier); Science Direct; MEDLINE/PubMed; Web of Science; Cumulative Index to Nursing and Allied Health Literature (CINAHL); Cochrane; and Virtual Health Library (VHL). It should be noted that an advanced search was carried out in each database. The search was carried out with the help of content accessed by the *Universidade Federal do Rio Grande do Norte* (UFRN), via the Federated Academic Community (CAFe - *Comunidade Acadêmica Federada*), through the Coordination for the Improvement of Higher Education Personnel (CAPES - *Coordenação de Aperfeiçoamento de Pessoal de Nível Superior*).

As a study search strategy, the following descriptors were defined, ordered in Health Sciences Descriptors (DeCS - *Descritores em Ciências da Saúde*) and Medical Subject Headings (MeSH): 1# “Venous Insufficiency”, 2# “Lower Extremity”, 3# “Leg”. Moreover, the keyword 4# “Peripheral” was used in the search. The search strategies in each database were performed according to Chart 1.

**Chart 1:** Search strategies defined in each selected database, Natal, Rio Grande do Norte, Brazil, 2019

Database	Search strategy
Scopus	ALL(“Venous Insufficiency”) AND (“Lower Extremity”) ALL(“Venous Insufficiency”) AND (“Leg”) ALL(“Venous Insufficiency”) AND (“Peripheral”) ALL(“Venous Insufficiency”) AND (“Lower Extremity”) AND (“Leg”) ALL(“Venous Insufficiency”) AND (“Lower Extremity”) AND (“Peripheral”)
Science Direct	(“Venous Insufficiency”) AND (“Lower Extremity”) (“Venous Insufficiency”) AND (“Leg”) (“Venous Insufficiency”) AND (“Peripheral”) (“Venous Insufficiency”) AND (“Lower Extremity”) AND (“Leg”) (“Venous Insufficiency”) AND (“Lower Extremity”) AND (“Peripheral”)
MEDLINE/PubMed	(“Venous Insufficiency”) AND (“Lower Extremity”) (“Venous Insufficiency”) AND (“Leg”) (“Venous Insufficiency”) AND (“Peripheral”) (“Venous Insufficiency”) AND (“Lower Extremity”) AND (“Leg”) (“Venous Insufficiency”) AND (“Lower Extremity”) AND (“Peripheral”)
Web of Science	((ALL=(“Venous Insufficiency”) AND (“Lower Extremity”) ((ALL=(“Venous Insufficiency”) AND (“Leg”) ((ALL=(“Venous Insufficiency”) AND (“Peripheral”) ((ALL=(“Venous Insufficiency”) AND (“Lower Extremity”) AND (“Leg”) ((ALL=(“Venous Insufficiency”) AND (“Lower Extremity”) AND (“Peripheral”)
CINAHL	(“Venous Insufficiency”) AND (“Lower Extremity”) (“Venous Insufficiency”) AND (“Leg”) (“Venous Insufficiency”) AND (“Peripheral”) (“Venous Insufficiency”) AND (“Lower Extremity”) AND (“Leg”) (“Venous Insufficiency”) AND (“Lower Extremity”) AND (“Peripheral”)
Cochrane	(“Venous Insufficiency”) AND (“Lower Extremity”) (“Venous Insufficiency”) AND (“Leg”) (“Venous Insufficiency”) AND (“Peripheral”) (“Venous Insufficiency”) AND (“Lower Extremity”) AND (“Leg”) (“Venous Insufficiency”) AND (“Lower Extremity”) AND (“Peripheral”)
Virtual Health Library	(“Venous Insufficiency”) AND (“Lower Extremity”) (“Venous Insufficiency”) AND (“Leg”) (“Venous Insufficiency”) AND (“Peripheral”) (“Venous Insufficiency”) AND (“Lower Extremity”) AND (“Leg”) (“Venous Insufficiency”) AND (“Lower Extremity”) AND (“Peripheral”)

Regarding the inclusion and exclusion criteria, studies that addressed the research questions, studies available in applied databases and studies published in Portuguese, English or Spanish were included. Editorials, letters to the editor, abstracts of presentations at scientific events and expert opinions were excluded. It should be noted that a time frame was not made so that the search for literature to be explored would be as broad as possible. In addition, book chapters were considered in the sample, in view of their important conceptual aspects for analysis.

### Data organization and analysis

The search was carried out between February and June 2019 by two researchers, independently, on the same days and times, using different computers and following the search and data extraction protocol. The studies were analyzed by dynamically reading the titles and abstracts, and then reading them in their entirety. Studies that did not fulfill the eligibility criteria were excluded, duplicates were counted only once, and disagreements among reviewers were resolved by consensus.

The mapping and data extraction process was based on an instrument containing information about: publication identification (identification number, study title, indexed database, file type, year, country and publication language); methodological aspects (methodology used and type of approach), and aspects related to concept analysis, such as attributes, antecedents and consequences, conceptual definition of consequence, and operational definition of consequence (empirical references); and at-risk population and associated conditions.

The results were descriptively presented in charts and tables.

## RESULTS

**Figure 1:**
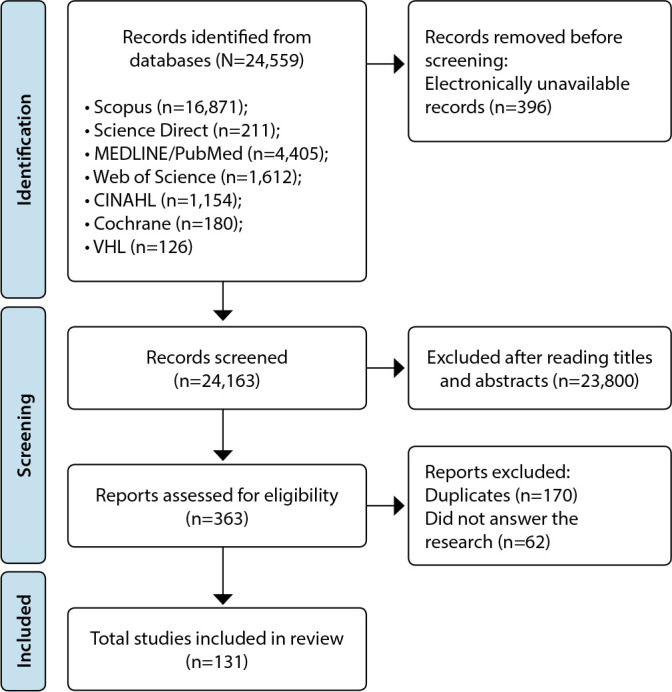
Flowchart of the study selection process, Natal, Rio Grande do Norte, Brazil, 2019

Searches in Scopus (Elsevier) found 16,871 studies, in Science Direct, 211, in MEDLINE/PubMed, 4,405, in Web of Science, 1,612, in CINAHL, 1,154, in Cochrane, 180, and in VHL, 126, totaling 24,559. Of these, 396 were electronically unavailable. After analyzing the titles and abstracts, 23,800 were excluded because they did not fulfill the eligibility criteria, and 170 were counted only once because they were duplicates. Thus, 193 studies were read in full, of which 131 studies made up the final sample (126 articles, 3 books chapters and 2 care protocols). Figure 1 shows the search flowchart in the databases and study selection.

According to the identified data, most of the studies were published between 2015 and 2019, accounting for 32.8% (n=43) of the studies. Regarding the method, the reviews were based on evidence (44.2%; n=58). The quantitative approach prevailed among the studies (79.3%; n=104). The continent with the most publications was North America, with 48.8% (n=64), and the language was English, with 96.1% (n=126).

Chart 2 shows a synthesis of conceptual definitions for “Impaired Peripheral Venous Return”. The expression is used in studies on vascular disorders, notably venous insufficiency. The most common definitions are related to functional impairment or dysfunction.

**Chart 2:** Synthesis of the most recent conceptual definitions of Impaired Peripheral Venous Return, Natal, Rio Grande do Norte, Brazil, 2019

Author(s)/Year	Study	Definition
Novak et al. (2019)^(22)^	Current therapeutic interventions in lower extremity venous insufficiency: a comprehensive review	A condition developed when the return of peripheral blood is impaired.
Ekici, Kartal, & Ferhatoglu (2019)^(23)^	Association between hemorrhoids and lower extremity chronic venous insufficiency	A congenital or acquired impairment of blood flow back to the heart.
Pieper et al. (2009)^(24)^	Impact of injection drug use on distribution and severity of chronic venous disorders	Reduction of the venous flow from the legs to the heart.

The common attribute identified was “decreased venous flow”, found in 117 studies (89.3% of the sample). Therefore, the definition of the diagnostic proposal was “decreased peripheral venous flow directed to the heart”.

Table 1 shows the antecedents and consequences of impaired peripheral venous return. The antecedents most frequently found were “structural and/or functional valve deficiency”, “advanced age” and “peripheral venous thrombosis”. The most common consequences were “peripheral edema”, “venous ulcer” and “pain in the extremity”.

**Table 1:** Antecedents (n=378) and consequences (n=339) of Impaired Peripheral Venous Return

	n (%)
Antecedents	
Individuals with structural and/or functional valve deficiency	57 (43.5)
Older adults	51 (38.9)
Peripheral venous thrombosis	43 (38.9)
Obesity	39 (29.8)
Female gender	37 (28.2)
Individuals with dysfunctional muscle propulsion	32 (24.4)
Prolonged time sitting or standing	31 (23.7)
Heredity	30 (22.9)
Pregnant women	28 (21.4)
Sedentary lifestyle	16 (12.2)
Reduced ankle mobility	6 (4.6)
Heart failure	5 (3.8)
Kidney injury	3 (2.2)
Consequences	
Signs	
Peripheral edema	72 (55.0)
Venous ulcer	64 (48.9)
Altered skin color	53 (40.5)
Varicose veins	49 (37.4)
Symptoms	
Pain in the extremity	41 (31.3)
Muscle fatigue	29 (22.1)
Paresthesia	26 (19.9)
Decreased perceived quality of life	5 (3.8)

The diagnostic label proposal was structured using the following NANDA-I axes: Diagnostic focus: venous return; Diagnostic subject: individual; Judgment: impaired; Location: peripheral; Diagnosis category: problem-focused diagnosis. The diagnostic content consisted of eight defining characteristics, five related factors, six at-risk populations and four associated conditions (Chart 3).

**Chart 3:** Proposed structure for the nursing diagnosis “Impaired Peripheral Venous Return”, based on concept analysis

Domain 4 - Activity/rest Class 4 - Cardiovascular/pulmonary responses Impaired peripheral venous return	
Definition	
Decreased peripheral venous flow directed to the heart	
**Defining characteristics**	
Signs Peripheral edema	Symptoms Pain in the extremity
Venous ulcer	Muscle fatigue
Altered skin color	Paresthesia
Varicose veins	Decreased perceived quality of life
**Related factors**	
Long-term obesity	
Sitting	
Prolonged standing	
Reduced ankle mobility	
Sedentary lifestyle	
**At-risk populations**	
Older women Individuals with hereditary predisposition Pregnant women Individuals with dysfunctional muscle propulsion Individuals with structural and/or functional valve deficiency	
**Associated conditions**	
Heart failure	
Kidney injury	
Peripheral venous thrombosis	
Venous insufficiency	

## DISCUSSION

According to the International Council of Nurses, autonomous and collaborative nursing care requires assessment of individuals’ responses to their health status (International Council of Nurses (n.d.). Although nurses have been intervening on conditions related to impaired venous return/venous stasis^(6-11)^, their unique function of assessing human responses might not have been evident due to the lack of a standardized nursing diagnosis.

Furthermore, nurses’ clinical reasoning toward impaired venous returns as a phenomenon of concern, which might lead to complications such as varicose veins and DVT, might require improvement^(25-26)^. The structure of NANDA-I diagnoses facilitates clinical reasoning by providing potential causes and cues for the diagnostic focus. In this study, it was developed a diagnostic proposal, “Impaired Peripheral Venous Return”, in order to address a gap in the NANDA-I classification.

Over 130 studies published in several continents were used to address critical attributes, antecedents and consequences of the phenomenon. “The impairment of venous flow toward the heart”, a critical attribute of “Impaired Peripheral Venous Return”, depicts a flow decrease due to the antecedent elements of structural and/or functional valve deficiency^(27)^ or a dysfunctional propulsion by calf muscles - the soleus and gastrocnemius muscles^(28)^. The antecedent elements of this phenomenon and their cues are discussed below.

Individuals at an advanced age are important at-risk populations. Older adults comprise an important risk group for CVD. Age is an important factor that can contribute to venous return impairment due to activity limitation as a result of loss of bone and muscle mass, vascular stiffness, reduced strength and flexibility, decreased aerobic capacity and gait alterations, which may lead to prolonged time sitting^(29-30)^.

During walking, about 25 mL of blood is discharged from the plantar veins with each step^(31)^. Also, 60-90 mL of blood is moved upwards from intermuscular and intramuscular veins by calf contraction^(32)^. Therefore, prolonged sitting, prolonged standing and reduced ankle mobility can contribute to impaired venous return. Furthermore, individuals with dysfunctional muscle propulsion and individuals with structural and/or functional valve deficiency are populations that must be carefully assessed for this condition. Having an active lifestyle is beneficial to reduce lower limb edema and to promote hemodynamic muscle improvement by strengthening the muscles and stimulating the calf muscle pump so that blood overcomes gravity toward the heart^(33)^.

The genetic predisposition associated with environmental stimuli are important conditions for the development of cardiovascular changes^(34)^. Individuals with hereditary propensity have a predisposing factor for developing problems in venous return. Likewise, obesity is a condition that occurs with great frequency associated with other clinical conditions. Thus, it favors the occurrence of cardiovascular events that can cause problems in venous return.

During normal pregnancy, veins in general are exposed to relaxation of the muscle walls by progesterone and to elevated blood pressure induced by an increased blood volume. In addition to this, lower limb veins are subjected to increased pressure by the growing uterus, which pressures the pelvic veins and the inferior vena cava^(35)^. In fact, a reduction in the peak systolic velocity of the popliteal vein has been found from the 25^th^ to the 35^th^ week of pregnancy^(36)^. Therefore, pregnant women are at increased risk for impaired venous return.

One of the major associated conditions is peripheral venous thrombosis, a clinical condition characterized by a blood clot in a deep vein, especially those located in the lower limb, in addition to a valve degeneration and, consequently, greater difficulty in transporting blood to the heart^(37)^. Regarding the consequences of the concept, edema occurs as a sign of fluid accumulation in the lower limbs^(38)^, which might lead to pain in the extremity, a symptom generally described as throbbing, a heaviness or a feeling of pressure, associated with discomfort^(39)^. As the antecedents interact with individuals continuously, an increasing number of consequences may occur, along with an exacerbation of their clinical spectrum, resulting in a greater degree of impairment^(40)^. Therefore, other consequences, such as muscle fatigue, paresthesia, and venous ulcer, may follow venous edema and pain, in more advanced cases of impaired peripheral venous return, unless interventions are implemented.

In addition to physical implications, it is also observed an association of the negative impact of this nursing diagnosis on the affected population’s mental health. This fact is evidenced in the identification of impaired quality of life as defining characteristics of the present diagnosis. Venous disease directly affects socioeconomic levels, and may limit the performance of individuals’ daily activities, from simple activities, to removing them from their work activities^(41)^.

Given the above, it is believed that nurses can play an important role in establishing an early and accurate diagnosis of impaired peripheral venous return during the provision of care. The inclusion of a routine assessment of venous return can prevent unnoticed problems as well as the later complications that are likely to occur and, consequently, ensure an enhancement in patient’ quality of life.

### Study limitations

The results of this study are limited by language restrictions applied to the literature review. Furthermore, it did not assess the studies’ methodological quality, and included other previous reviews. However, several methods were necessary to synthesize definitions and identify the broader uses of the concepts. Finally, studies focused on content and clinical validity of the diagnostic proposal are required.

### Contributions to nursing, health and public policies

Developing a nursing diagnosis proposal focusing on venous return within the structure proposed by NANDA-I will facilitate clinical reasoning and decision making by both practitioners and students, thus supporting future research on diagnostic accuracy and intervention effectiveness. It will also help nurses articulate their unique body of knowledge within the interdisciplinary context when delivering care to patients with venous return alterations.

## CONCLUSIONS

Critical attributes, antecedent and consequent elements of “Impaired Peripheral Venous Return” were determined in concept analysis, including a robust literature review. The formulated nursing diagnosis was proposed as part of Domain 4, Activity/rest, in Class 4, Cardiovascular/pulmonary responses, with eight defining characteristics, five related factors, six at-risk populations and four associated conditions.

The new diagnostic proposal developed within the NANDA-I structure, supported at the level of evidence 2.1.1, may provide early identification of the problem and guide a more targeted nursing care, with a view to reducing risks to patients. This proposal will be submitted to the NANDA-I Diagnostic Development Committee.

## References

[1] World Health Organization (WHO) (2017). Cardiovascular Diseases.

[2] World Health Organization (WHO) (2013). Global action plan for the prevention and control of noncommunicable diseases 2013-2020.

[3] Tracz E, Zamojska E, Modrzejewski A, Zaborski D, Grzesiak W. (2015). Quality of life in patients with venous stasis ulcers and others with advanced venous insufficiency. Holist Nurs Pract.

[4] Branisteanu DE, Feodor T, Baila S, Mitea IA, Vittos O. (2019). Impact of chronic venous disease on quality of life: results of vein alarm study. Exp Ther Med.

[5] Lumley E, Phillips P, Aber A, Buckley-Woods H, Jones GL, Michaels JA. (2019). Experiences of living with varicose veins: a systematic review of qualitative research. J Clin Nurs.

[6] Thompson A, Walter S, Brunton LR, Pickering GT, Mehendale S, Smith AJ (2011). Anti-embolism stockings and proximal indentation. Br J Nurs.

[7] Badger J, Taylor P, Papworth N, Swain I. (2018). Electrical stimulation devices for the prevention of venous thromboembolism: preliminary studies of physiological efficacy and user satisfaction. J Rehabil Assist Technol Eng.

[8] Pi H, Ku H, Zhao T, Wang J, Fu Y. (2018). Influence of ankle active dorsiflexion movement guided by inspiration on the venous return from the lower limbs: a prospective study. J Nurs Res.

[9] Benigni JP, Uhl JF, Balet F, Filori P, Chahim M. (2018). Evaluation of three different devices to reduce stasis edema in poorly mobile nursing home patients. Int Angiol.

[10] Gonzalez A. (2017). The effect of a patient education intervention on knowledge and venous ulcer recurrence: results of a prospective intervention and retrospective analysis. Ostomy Wound Manage.

[11] Probst S, Allet L, Depeyre J, Colin S, Skinner MB. (2019). A targeted interprofessional educational intervention to address therapeutic adherence of venous leg ulcer persons (TIEIVLU): study protocol for a randomized controlled trial. Trials.

[12] Flanagan J. (2018). Regarding nursing languages: moving beyond how we feel. Int J Nurs Knowl.

[13] Herdman TH, Kamitsuru S. (2018). NANDA-I Nursing diagnoses: definitions and classification 2018-2020.

[14] Tansey EA, Montgomery LEA, Quinn JG, Roe SM, Johnson CD. (2019). Understanding basic vein physiology and venous blood pressure through simple physical assessments. Adv Physiol Educ.

[15] Lopes MV, Silva VM, Araujo TL. (2012). Methods for establishing the accuracy of clinical indicators in predicting nursing diagnoses. Int J Nurs Knowl.

[16] Carvalho EC, Cruz DA, Herdman TH. (2013). Contribution of standardized languages for knowledge production, clinical reasoning and clinical nursing practice. Rev Bras Enferm.

[17] Herdman TH, Kamitsuru S, Lopes CT. (2021). NANDA-I Nursing diagnoses: definitions and classification 2021-2023.

[18] Walker LO, Avant KC. (2019). Strategies for theory construction in nursing.

[19] Page MJ, McKenzie JE, Bossuyt PM, Boutron I, Hoffmann TC, Mulrow CD (2021). The PRISMA 2020 statement: an updated guideline for reporting systematic reviews. Int J Surg.

[20] Whittemore R, Knafl K. (2005). The integrative review: updated methodology. J Adv Nurs.

[21] Peters MDJ, Godfrey C, McInerney P, Munn Z, Tricco AC, Khalil H., Aromataris E, Munn Z (2020). Chapter 11: Scoping Reviews (2020 version).

[22] Novak CJ, Khimani N, Kaye AD, Yong RJ, Urman RD. (2019). Current therapeutic interventions in lower extremity venous insufficiency: a comprehensive review. Curr Pain Headache Rep.

[23] Ekici U, Kartal A, Ferhatoglu MF. (2019). Association Between Hemorrhoids and Lower Extremity Chronic Venous Insufficiency. Cureus.

[24] Pieper B, Templin TN, Kirsner RS, Birk TJ. (2009). Impact of injection drug use on distribution and severity of chronic venous disorders. Wound Repair Regen.

[25] Davies AH. (2019). The seriousness of chronic venous disease: a review of real-world evidence. Adv Ther.

[26] Silva JS, Lee J, Grisante DL, Lopes JL, Lopes CT. (2020). Conhecimento, avaliação de risco e autoeficácia quanto a tromboembolismo venoso entre enfermeiros. Acta Paul Enferm.

[27] Oh H, Boo S, Lee JA. (2017). Clinical nurses' knowledge and practice of venous thromboembolism risk assessment and prevention in South Korea: a cross-sectional survey. J Clin Nurs.

[28] Mutlak O, Aslam M, Standfield NJ. (2019). Chronic venous insufficiency: a new concept to understand pathophysiology at the microvascular level: a pilot study. Perfusion.

[29] Attaran RR. (2018). Latest innovations in the treatment of Venous disease. J Clin Med.

[30] Dai X, Hummel SL, Salazar JB, Taffet GE, Zieman S, Schwartz JB. (2015). Cardiovascular physiology in the older adults. J Geriatr Cardiol.

[31] Colón CJP, Molina-Vicenty IL, Frontera-Rodríguez M. (2018). Muscle and bone mass loss in the elderly population: advances in diagnosis and treatment. J Biomed (Syd).

[32] Uhl JF, Gillot C. (2012). Anatomy of the foot venous pump: physiology and influence on chronic venous disease. Phlebology.

[33] Li T, Yang S, Hu F, Geng Q, Lu Q, Ding J. (2020). Effects of ankle pump exercise frequency on venous hemodynamics of the lower limb. Clin Hemorheol Microcirc.

[34] Pereira JF. (2019). Plataforma ativa de apoio plantar para ambiente de trabalho: avaliação dos movimentos e estímulos favoráveis ao retorno venoso.

[35] Seidel AC, Coelho RL, Coelho ML, Belczak CEQ. (2014). É a lesão venosa a única responsável pela clínica da insuficiência venosa crônica dos membros inferiores?. J Vasc Bras.

[36] Smyth RM, Aflaifel N, Bamigboye AA. (2015). Interventions for varicose veins and leg oedema in pregnancy. Cochrane Database Syst Rev.

[37] Gimunová M, Zvonař M, Kolářová K, Janík Z, Mikeska O, Musil R (2017). Changes in lower extremity blood flow during advancing phases of pregnancy and the effects of special footwear. J Vasc Bras.

[38] Min SK, Kim YH, Joh JH, Kang JM, Park UJ, Kim HK (2016). Diagnosis and Treatment of Lower Extremity Deep Vein Thrombosis: Korean Practice Guidelines. Vasc Specialist Int.

[39] Ratchford EV, Evans NS. (2017). Approach to Lower Extremity Edema. Curr Treat Options Cardiovasc Med.

[40] Youn YJ, Lee J. (2019). Chronic venous insufficiency and varicose veins of the lower extremities. Korean J Intern Med.

[41] Miraj AKA, Rahaman HNA, Rahman MM, Khan MSU. (2020). Impact of leg ulcers on quality of life: financial, social, and psychological implications among the patients attending OPD of vascular surgery: a study in Bangabandhu Sheikh Mujib Medical University, Dhaka, Bangfladesh. Br J Res.

